# Supplementary material for: How disconfirmatory evidence shapes confidence in decision-making
Source: Commun Psychol. 2025 Oct 29;3:150. doi: 10.1038/s44271-025-00325-3 (PMC12571896; doi:10.1038/s44271-025-00325-3)
Supplement: Supplementary file 2 — Supplementary Material [file 44271_2025_325_MOESM2_ESM.pdf]

Supplementary Information

How disconfirmatory evidence shapes confidence in decision-making

Annika Boldt*[1], Yishu Sun[1] & Kobe Desender[2]

1. Institute of Cognitive Neuroscience, UCL, London, UK
2. Brain and Cognition, KU Leuven, Leuven, Belgium

Corresponding author:

Dr. Annika Boldt, Institute of Cognitive Neuroscience, UCL, Alexandra House, 17 Queen Square, WC1N 3AZ, London, UK, +44 (0)20 7679 1199, a.boldt@ucl.ac.uk

Keywords:

confidence, metacognition, positive-evidence bias, robust averaging, decision making

Supplementary Note 1

Table S1 lists all studies with their participant and trial numbers. For convenience, we assigned a number to each dataset. The precise methods of each individual study can be found in their respective publication. Here, we only list the essential details in which the studies differed. Most data sets comprised 4 conditions from a 2 x 2 design, except for data sets 2 (Exp. 8; Boldt, 2015) and 5 (Exp. 3a; Desender et al., 2019a), which both contained 6 conditions from a 2 x 3 design with color variance varied in 3 levels. In data sets 7-9 the exact value of the low-mean condition was adjusted with an ongoing staircase, but the analysis was conducted after averaging across the data to form the distinct 4 difficulty conditions. Data sets 1-7 included speeded decisions, whereas data sets 8 and 9 included unspeeded decisions. Data sets 1-7 used a 6-point confidence scale ranging from "certainly wrong" to "certainly correct", whereas data sets 8-9 collected choices and confidence concurrently with a scale ranging from "sure red" via "guess" to "sure blue". The confidence data were hence later transformed to match the above format. More specifically, we had to transform the continuous confidence measurements from Desender et al. (2018; 2019b) to ordinal ratings ranging from 1-6 (Desender et al., 2019b) or 4-6 (Desender et al., 2018). EEG was recorded for data sets 2, 4 and 7, but these data are not further discussed here. For data sets 3 and 4, a predictive cue preceded all trials. For these two data sets, there were furthermore also "predicted confidence" trials (see Boldt et al., 2019), but these were not included in the present analysis. Data set 5 included trials which lay on the category boundary and where an objectively correct answer therefore did not exist. Those trials were excluded from the present analysis. Data sets 7-9 included an additional information seeking response. In all three data sets there were two conditions: a free-choice condition in which participants voluntarily decided whether they wanted to see an easier version of the stimulus before making their final choice and confidence, and a forced-choice condition in which participants were not given the option to see extra information and immediately provided a final choice and confidence. Because trials from the free-choice condition were contaminated by the additional evidence, these were excluded from the present analysis.

Table S1: Description of datasets included in the reanalysis.

Data set	N participants	Trials per participant	Reference
1	20	977 – 1023	Boldt, de Gardelle & Yeung (2017)
2	16	409 – 430	Boldt (2015); Exp. 8
3	17	715 – 839	Boldt, Schiffer, Waszak & Yeung (2019); Exp. 1
4	18	493 – 598	Boldt, Schiffer, Waszak & Yeung (2019); Exp. 2
5	11	352 – 376	Desender, Boldt, Verguts & Donner (2019a); Exp. 3a
6	9	249 – 256	Desender, Boldt, Verguts & Donner (2019a); Exp. 3b
7	15	154 – 160	Desender, Murphy, Boldt, Verguts & Yeung (2019b)
8	20	320	Desender, Boldt & Yeung (2018); Exp. 1
9	50	291 – 320	Desender, Boldt & Yeung (2018); Exp. 2
Sum	176	82118	

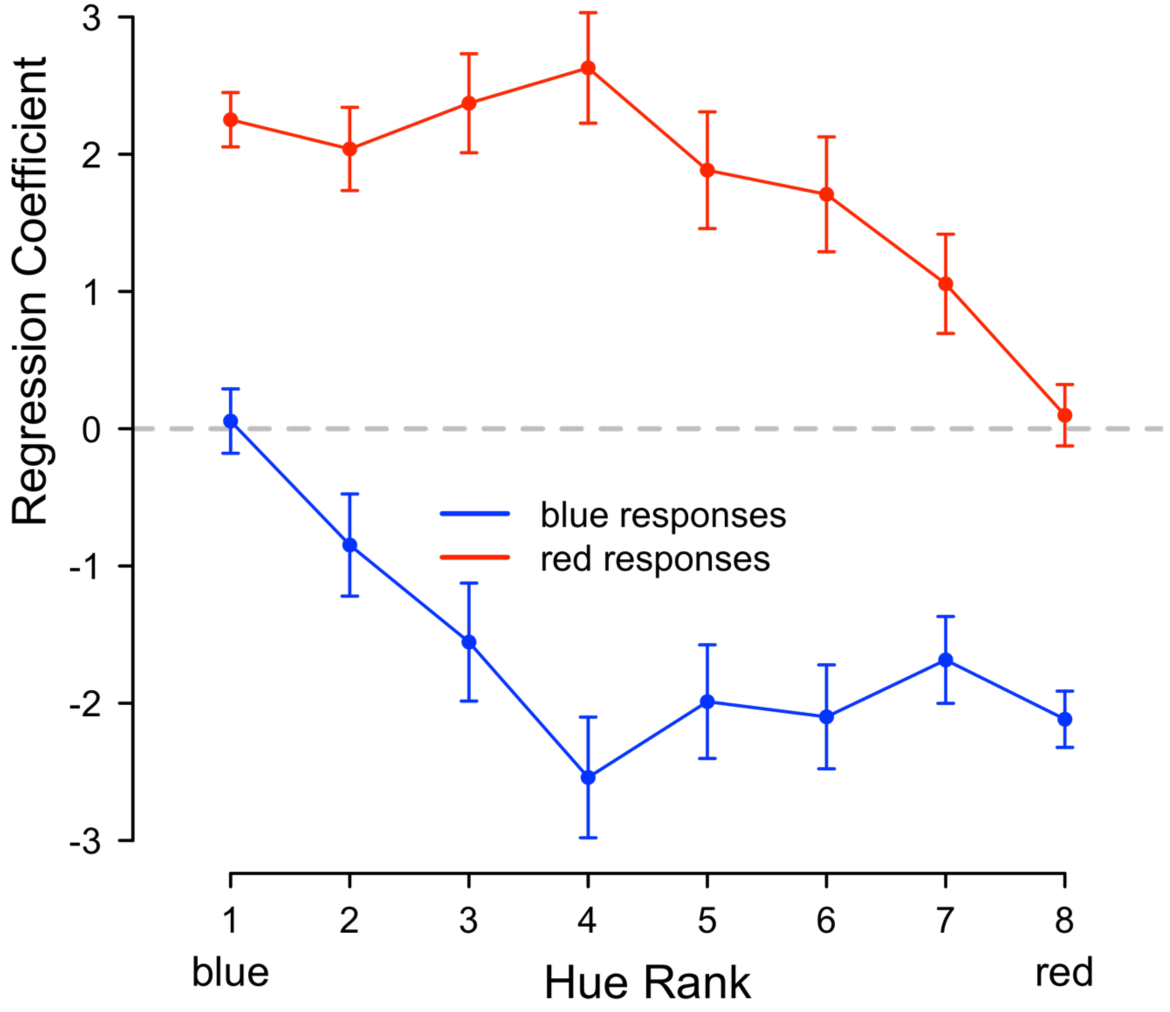

Figure S1. Confidence reliance effects split by response type. *Uncertainty for the empirical data is reflected in the error bars (2.5% and 97.5% Wald confidence intervals). The models were fit to all data including error trials, using the same mixed model approach as in the main text but replacing the split by stimulus category with response type. Post-hoc contrasts replicated the results pattern reported in the main text with a significant negative slope for red responses,* β = 22.73, *SE* = 0.57, *z* = 40.14, $p < 0.001$, *95% CI [21.38, 24.07], a positive slope for blue responses,* β = 20.47, *SE* = 0.60, *z* = 34.13, $p < 0.001$, *95% CI [19.05, 21.90], a stronger weight of incongruent elements compared to congruent elements, both for red responses,* β = 4.55, *SE* = 0.30, *z* = 15.31, $p < 0.001$, *95% CI [3.84, 5.25], and for blue responses,* β = 3.00, *SE* = 0.31, *z* = 9.84, $p < 0.001$, *95% CI [2.28, 3.73], and a stronger influence of inlying (positions 3-6) versus outlying (positions 1-2 and 7-8), both for red responses,* β = 5.31, *SE* = 0.49, *z* = 10.78, $p < 0.001$,

95% CI [4.14, 6.48], and blue responses, β = 6.06, SE = 0.51, z = 11.83, p < 0.001, 95% CI [4.84, 7.28].

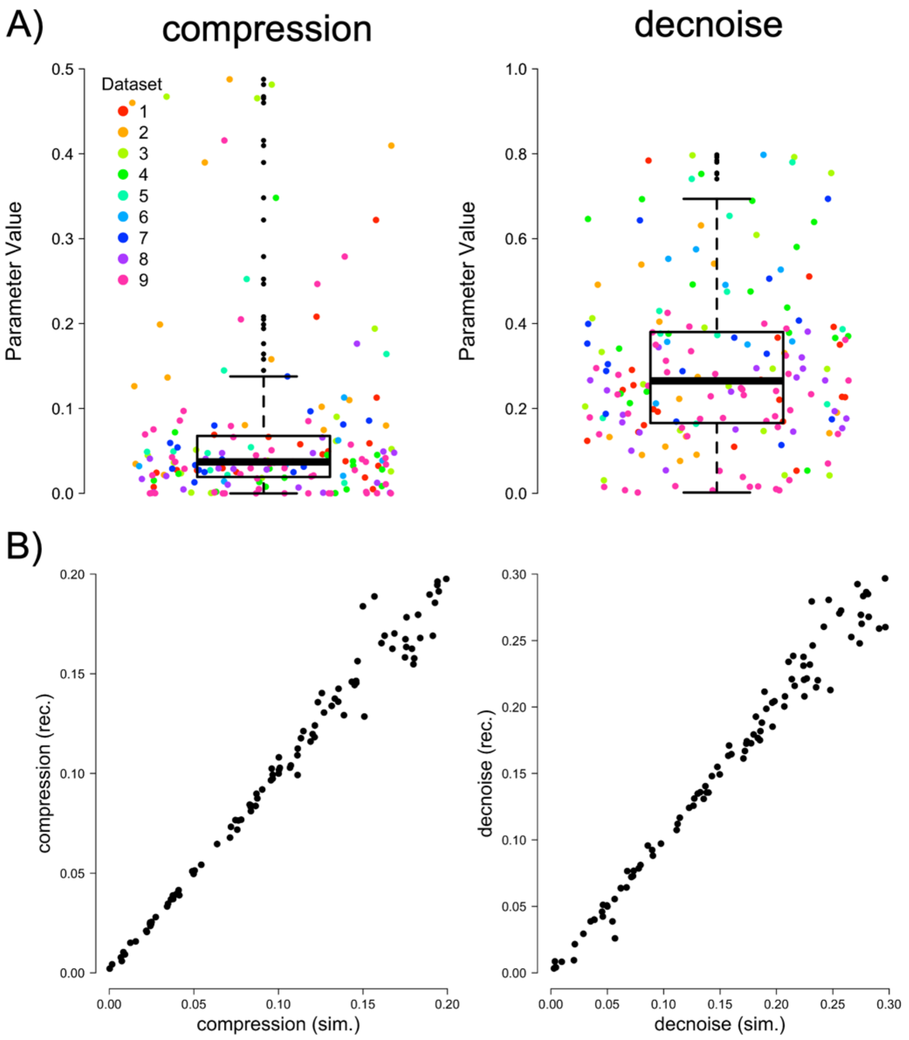

Figure S2. Parameters of the winning model (Model 4). *A) shows parameters distributions for the compression, decnoise, and metanoise parameters. The colors indicate the dataset. The superimposed boxplot shows the median and spans from the interquartile range with whiskers indicating the minimum and maximum values respectively and outliers represented as black dots. B) Depicts results from the parameter recovery (with N = 100 and assuming 250 trials per simulated dataset) for the winning model for the compression, dec-noise, and metanoise*

parameters. Simulated values (sim.) are shown on the x-axes and recovered values (rec.) on the y-axes.

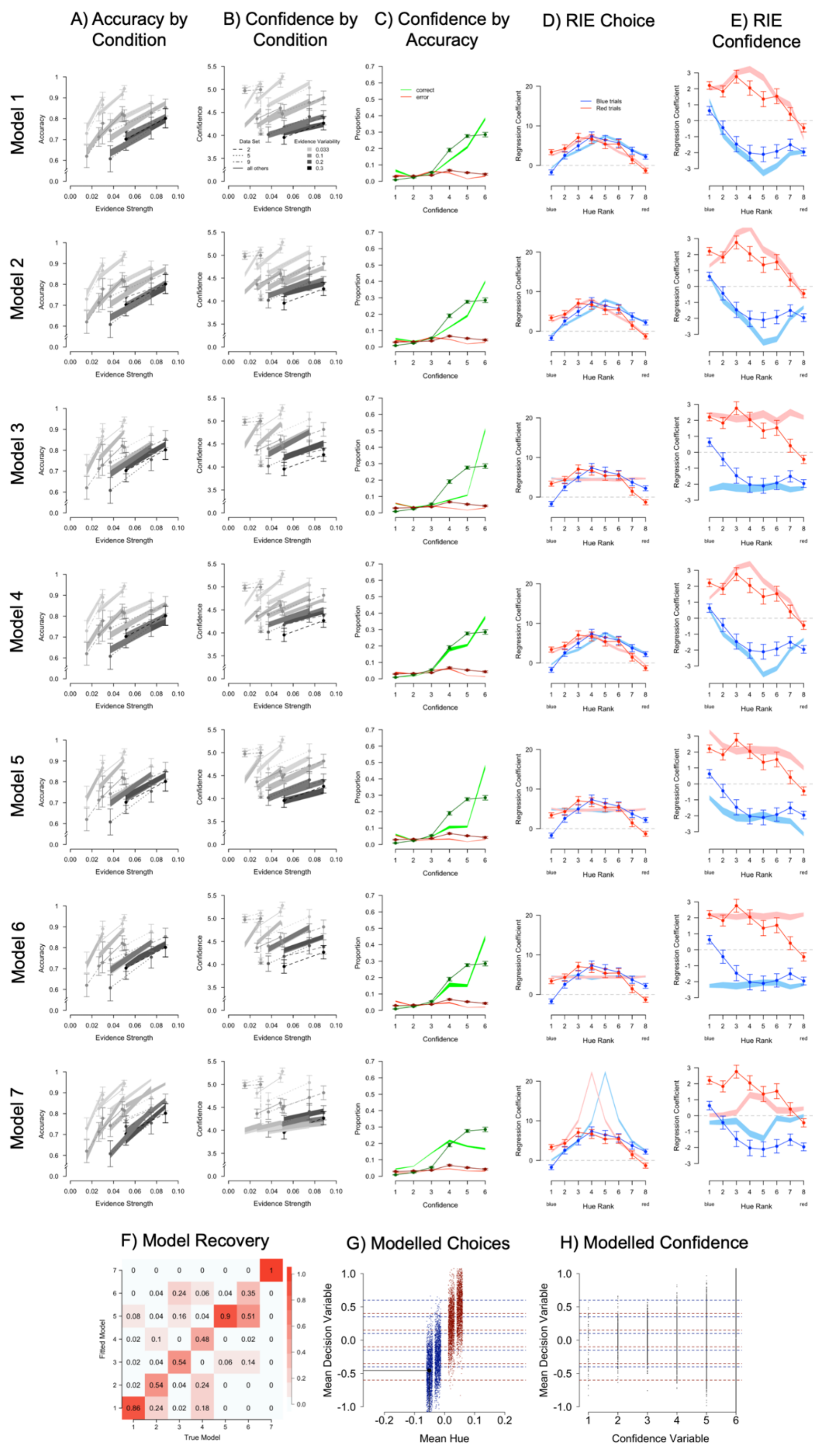
A) Accuracy by Condition
B) Confidence by Condition
C) Confidence by Accuracy
D) RIE Choice
E) RIE Confidence
Model 1
Model 2
Model 3
Model 4
Model 5
Model 6
Model 7
Accuracy
Evidence Strength
Confidence
Data Set
Evidence Variability
all others
Proportion
correct
error
Regression Coefficient
Blue trials
Red trials
Hue Rank
blue
red
F) Model Recovery
G) Modelled Choices
H) Modelled Confidence
Fitted Model
True Model
Mean Decision Variable
Mean Hue
Confidence Variable

Figure S3. Full behavioral and model findings. *Columns A-E depict the empirical lines and simulated bands for A) accuracy by condition, B) confidence by condition, C) confidence frequency by accuracy, D) choice reliance effects and E) confidence reliance effects. Uncertainty for the empirical data is reflected in the error bars (2.5% and 97.5% Wald confidence intervals). Uncertainty for the simulated data is reflected in the width of the bands (2.5% and 97.5% Wald confidence intervals). We collapsed across datasets as much as possible. Each row depicts the results for one model using the best-fitting parameter combinations for this respective candidate model. F) Model recovery matrix, showing the probability that data generated under a specific model is best accounted for by that same model (diagonal) versus the other models under consideration (off-diagonal). G-H) show the confidence extension of the log posterior ratio model (c-LPR) using data for the winning model (Model 4) with parameters from a single subject from Dataset 1. G) Stimuli in the experiment were sampled pseudorandomly, meaning that the average colors of the individual stimuli were kept in narrow tolerance bands around the 4 condition mean hue (x-axis). The y-axis depicts the mean decision variable resulting from robust averaging. Dashed horizontal lines reflect the confidence criteria for blue and red stimuli. The black dot reflects the stimulus example shown in Figure 2C). H) Transformation of the mean decision variable into confidence by means of the respective confidence criteria (blue and red horizontal dashed lines).*

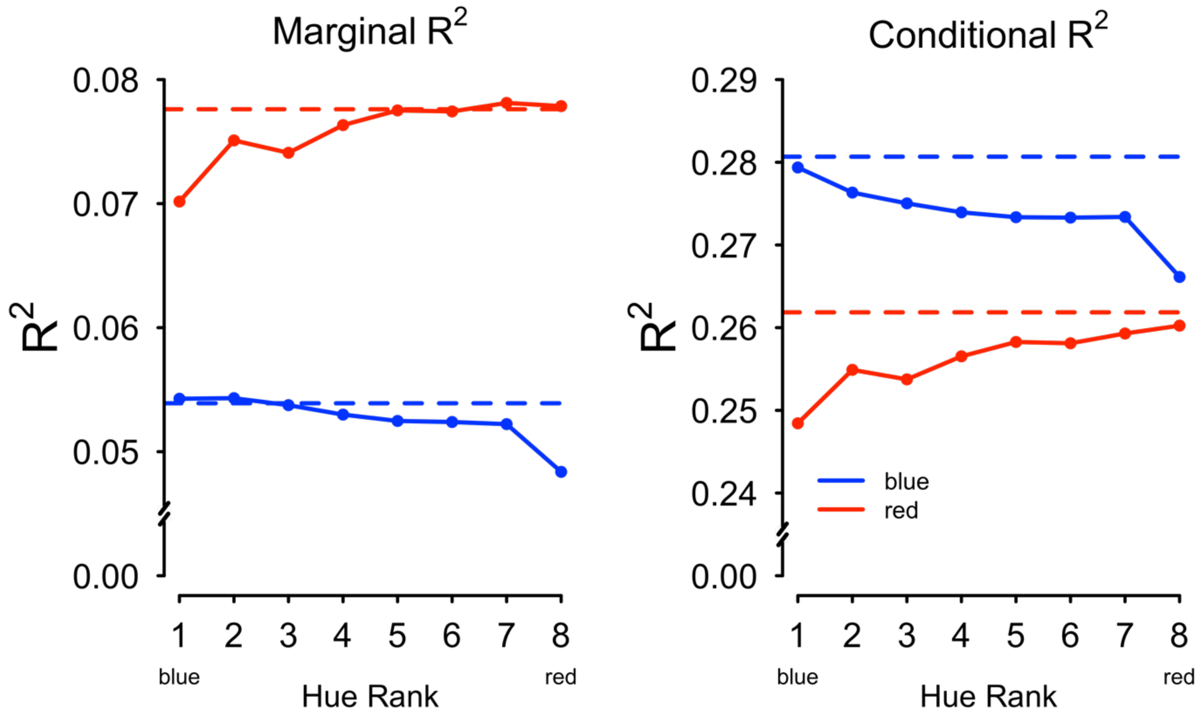

Figure S4. Converging evidence from stepwise regression analysis. *We conducted an additional analysis in which we calculated the* R^2 *for a mixed model predicting confidence from all eight items (dashed horizontal lines) and then eight additional models each leaving out one of the elements as a predictor. This process was repeated for both blue and red trials separately. In line with the main findings,* R^2 *drops more if category-incongruent evidence is removed from the regression. This pattern held true for both marginal* R^2 *(left panel) and conditional* R^2 *(right panel).* R^2 *were calculated using the R package MuMIn (Bartón, 2023).*

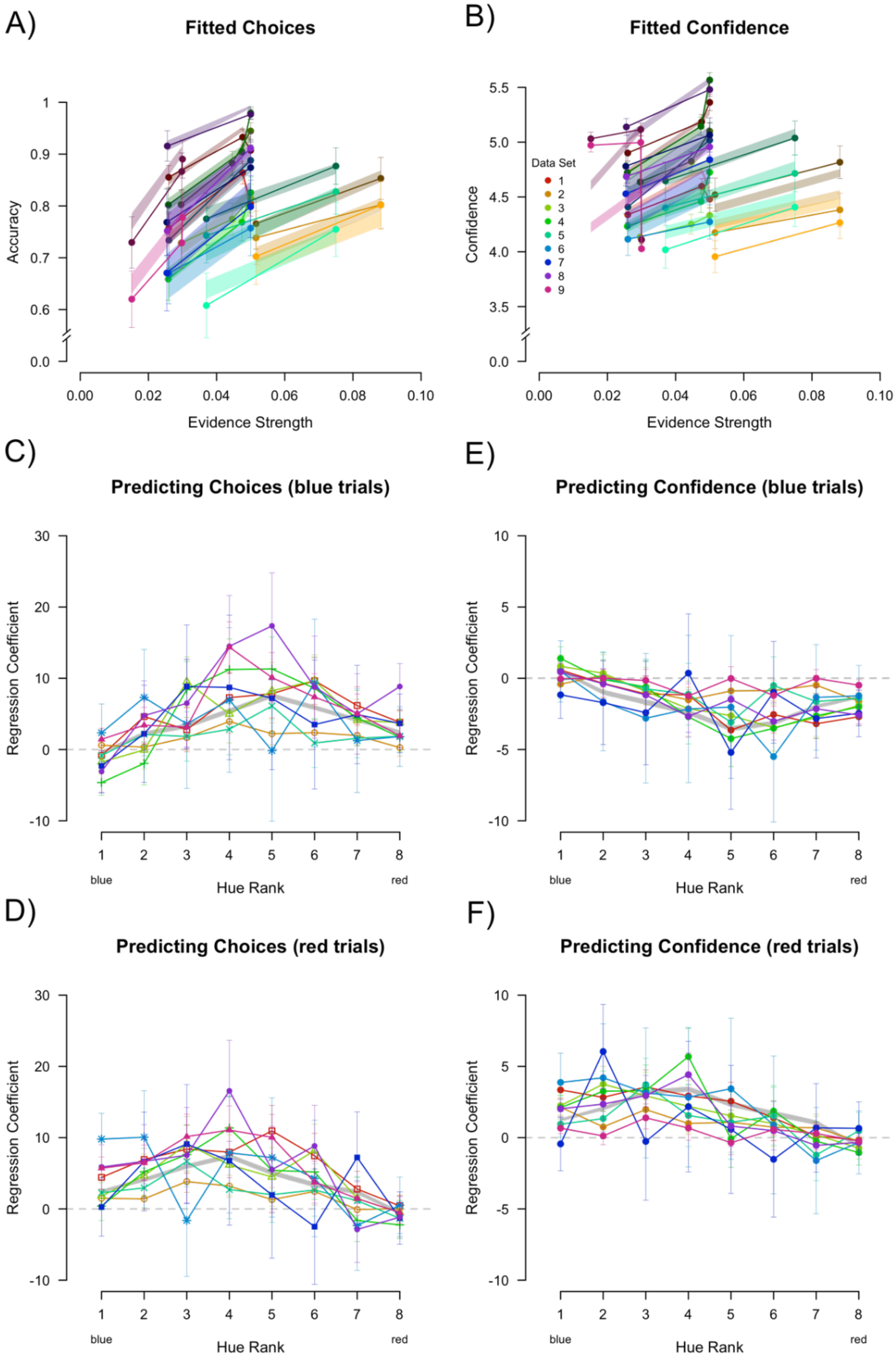
A)
Fitted Choices
Accuracy
Evidence Strength
B)
Fitted Confidence
Confidence
Data Set
1
2
3
4
5
6
7
8
9
Evidence Strength
C)
Predicting Choices (blue trials)
Regression Coefficient
Hue Rank
blue
red
E)
Predicting Confidence (blue trials)
Regression Coefficient
Hue Rank
blue
red
D)
Predicting Choices (red trials)
Regression Coefficient
Hue Rank
blue
red
F)
Predicting Confidence (red trials)
Regression Coefficient
Hue Rank
blue
red

Figure S5. Full behavioral and model findings for each dataset. *Panels A-F depict the empirical lines and simulated bands for A) accuracy by condition, B) confidence by condition, C) choice reliance effects, and D) confidence reliance effects. Uncertainty for the empirical data is reflected in the error bars (2.5% and 97.5% Wald confidence intervals). Uncertainty for the simulated data is reflected in the width of the bands (2.5% and 97.5% Wald confidence intervals). Each experiment is shown in a different color. Only results from the best-fitting model is shown here (Model 4) as colored bands (Panels A and B) or grey bands (Panels C-F). Please note that for Panels C-F, the grey band shows the estimate taken from the mixed models used to fit all data at once and hence there are no individual simulation results for each dataset.*

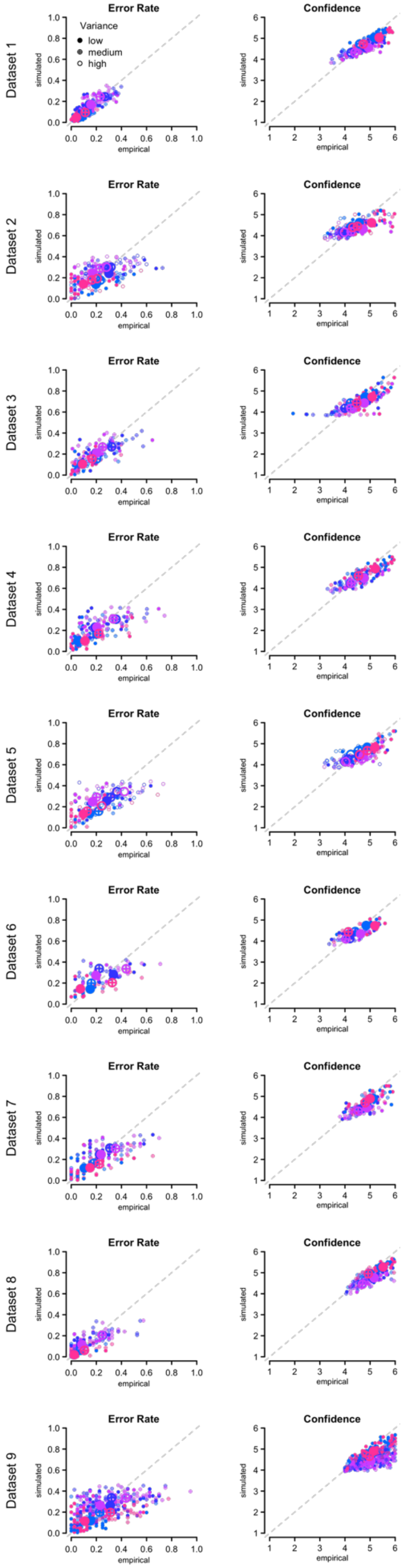
Error Rate
Confidence
Variance
low
medium
high
Dataset 1
Dataset 2
Dataset 3
Dataset 4
Dataset 5
Dataset 6
Dataset 7
Dataset 8
Dataset 9
simulated
empirical

113

Figure S6. Empirical and simulated error rates across datasets and participants. *Each large marker represents the average error rate (left panels) or confidence (right panels) for a given condition, with empirical values on the x-axis and simulated values on the y-axis. Marker color corresponds to the exaggerated hue used to represent the color mean condition, and marker fill indicates the variance condition. Smaller markers in matching colors show individual participants' average values for each condition. Each row of panels corresponds to a different dataset.*

Supplementary Note 2

Despite plans to match the Blue and Red Contexts and due to a coding mistake, the conditions significantly differed across all dependent variables. Error rates were higher in the Red Context, $t(31) = -7.88$, $p < .001$, $d_p = -1.39$, 95% CI [-1.88, -0.89]; a separate Bayesian paired-samples *t*-test yielded $BF_{10} = 1,848,168$, indicating extreme evidence for a difference. Response times were slower, $t(31) = -5.51$, $p < .001$, $d_p = -0.58$, 95% CI [-0.81, -0.35]; $BF_{10} = 3,947$, also indicating extreme evidence for a difference. Confidence was lower, $t(31) = 6.83$, $p < .001$, $d_p = 1.03$, 95% CI [0.66, 1.40]; $BF_{10} = 7.25 \times 10^{20}$, again providing extreme evidence in favor of the alternative hypothesis. For the confidence measure, the normality assumption was violated. To address this, we repeated the comparison using a Wilcoxon signed-rank exact test. The results remained significant ($V = 521$, $p < 0.001$). Please note that these three comparisons were specified as planned additional analyses in our preregistration. The condition difference was due to a coding mistake resulting in a red condition that was closer to the category boundary than intended.

Hypothesis 1: We predicted that confidence and accuracy would be linked meaningfully, in that confidence would be higher on correct than on error trials. We tested this hypothesis using a logistic mixed model in which single-trial accuracy was predicted by confidence. As expected, there was a significant fixed effect of confidence level, $\beta = 1.32$, $SE = 0.10$, $z = 12.99$, $p < 0.001$, 95% *CI* [1.12, 1.51]. This corresponds to an odds ratio of OR = 3.73, 95% CI [3.06, 4.55]: Mean accuracy was lowest for low confidence and highest for high confidence ($M_1 = 0.17$, $M_2 = 0.34$, $M_3 = 0.53$, $M_4 = 0.83$, $M_5 = 0.94$, $M_6 = 0.97$; see Figure S7). We can therefore conclude that participants used the confidence scale in a meaningful way.

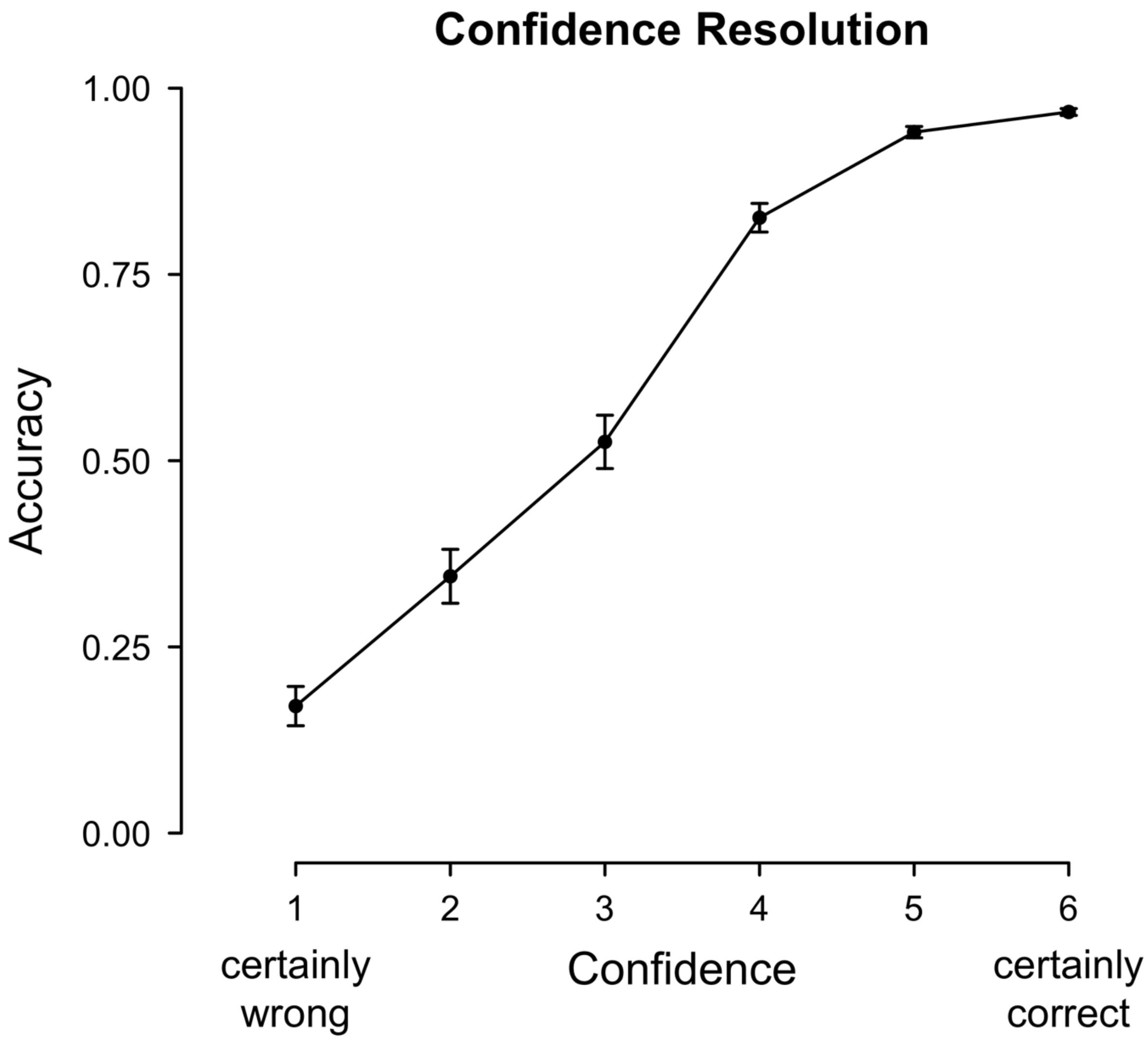

Figure S7. Confidence resolution. *Average accuracy plotted as a function of confidence level for Dataset 10. Uncertainty is reflected in the error bars (+/- 1 S.E.M.).*

Hypothesis 2: We first predicted to replicate the robust averaging effect for choices (De Gardelle & Summerfield, 2010), more specifically a stronger influence of the 'inlying' compared to the 'outlying' evidence samples (Hypothesis 2a). However, only for the red (β = 15.66, *SE* = 3.22, z = 4.87, p < 0.001, 95% CI [9.35, 21.96]) and blue trials (β = 9.38, *SE* = 4.10, z = 2.29, p = 0.022, 95% CI [1.35, 17.40]) did we find that the inlying positions (3, 4, 5, and 6) to have a larger absolute influence than the outlying positions (1, 2, 7, and 8). For the other two conditions, the post-hoc tests did not reach significance: for purple trials in the Red Context, β = 3.81, SE =

3.15, $z = 1.21$, $p = 0.227$, 95% CI [-2.37, 9.99]; and for purple trials in the Blue Context, β = -8.78, SE = 4.66, $z = -1.89$, $p = 0.059$, 95% CI [-17.90, 0.35], noting that the latter nonsignificant effect was actually in the opposite direction. Second, we tested for an effect of congruency comparing the 4 congruent with the 4 incongruent positions in post-hoc tests (Hypothesis 2b). Only conditions in the Red Context were significant, both for red trials (β = -15.60, SE = 6.40, $z = -2.44$, $p = 0.015$, 95% CI [-28.14, -3.05] and purple trails in the Red Context (β = 15.70, SE = 5.85, $z = 2.68$, $p = 0.007$, 95% CI [4.23, 27.16]). The other conditions in the Blue Context were not significant, neither the blue trials (β = -13.06, SE = 7.99, $z = -1.63$, $p = 0.102$, 95% CI [-28.73, 2.61]), not the purple trials in the Blue Context (β = 12.41, SE = 8.34, $z = 1.49$, $p = 0.137$, 95% CI [-3.94, 28.77]). Importantly, the red and blue trials numerically showed the opposite pattern of what we expected. We did not preregister a linear hypothesis, but to keep in parallel with the main text, we conducted it anyways. Only the purple trials in the Red Context were significant, β = 74.70, SE = 29.50, $z = 2.53$, $p = 0.011$, 95% CI [16.88, 132.51] (purple in Blue Context: β = 59.96, SE = 42.37, $z = 1.42$, $p = 0.157$, 95% CI [-23.10, 143.01]; blue: β = -60.36, SE = 38.55, $z = -1.57$, $p = 0.117$, 95% CI [-135.91, 15.19]; red: β = -39.35, SE = 30.61, $z = -1.29$, $p = 0.199$, 95% CI [-99.35, 20.64]). See also the left panels of Figure S7. It should be noted that we deviated slightly from the preregistration: although we had originally planned to fit all data within a single model, concerns about multicollinearity led us to instead fit four separate models (one for each color and context condition) rather than using dummy variables. The full statistical results from these models are listed in Table S2.

Table S2: Results from four logistic mixed models predicting trial-by-trial choices for the empirical data. SE = standard error; HR = hue rank; CI = confidence interval. Due to the small and unscaled range of the predictors, odds ratios are not directly interpretable and therefore not included.

Model	Predictor	β [95% CI]	SE	*z*	*p*
Blue trials	Intercept	-15.84 [-22.38, -9.29]	3.34	-4.74	< 0.001
	HR 1	4.70 [0.11, 9.28]	2.34	2.01	0.045
	HR 2	6.71 [2.32, 11.10]	2.24	3.00	0.003
	HR 3	10.90 [5.83, 15.97]	2.59	4.22	< 0.001

	HR 4	4.16 [-1.17, 9.49]	2.72	1.53	0.126
	HR 5	4.55 [-1.07, 10.16]	2.86	1.59	0.112
	HR 6	5.02 [0.33, 9.71]	2.39	2.10	0.036
	HR 7	1.79 [-2.79, 6.37]	2.34	0.77	0.443
	HR 8	2.05 [-2.49, 6.60]	2.32	0.89	0.376
Purple trials in blue context	Intercept	-15.21 [-24.15, -6.26]	4.56	-3.33	< 0.001
	HR 1	10.04 [5.10, 14.98]	2.52	3.99	< 0.001
	HR 2	5.57 [0.82, 10.32]	2.42	2.30	0.021
	HR 3	7.39 [2.17, 12.62]	2.67	2.77	0.0056
	HR 4	3.73 [-1.88, 9.34]	2.86	1.30	0.193
	HR 5	3.36 [-2.62, 9.35]	3.05	1.10	0.271
	HR 6	1.65 [-3.74, 7.04]	2.75	0.60	0.548
	HR 7	4.68 [-0.17, 9.53]	2.47	1.89	0.059
	HR 8	4.62 [-0.42, 9.67]	2.57	1.80	0.072
Purple trials in red context	Intercept	-22.69 [-29.10, -16.27]	3.27	-6.93	< 0.001
	HR 1	1.77 [-1.64, 5.18]	1.74	1.02	0.308
	HR 2	4.04 [0.70, 7.38]	1.70	2.37	0.018
	HR 3	0.94 [-2.73, 4.61]	1.87	0.50	0.616
	HR 4	6.73 [2.75, 10.72]	2.03	3.31	< 0.001
	HR 5	5.68 [1.48, 9.89]	2.14	2.65	0.008
	HR 6	9.88 [6.27, 13.50]	1.84	5.36	< 0.001
	HR 7	6.89 [3.54, 10.24]	1.71	4.04	< 0.001
	HR 8	6.72 [3.16, 10.28]	1.82	3.70	< 0.001
Red trials	Intercept	-17.01 [-24.44, -9.59]	3.79	-4.49	< 0.001
	HR 1	2.26 [-1.33, 5.85]	1.83	1.24	0.217

	HR 2	0.61 [-2.97, 4.18]	1.83	0.33	0.739
	HR 3	2.93 [-0.77, 6.63]	1.89	1.55	0.121
	HR 4	3.22 [-1.34, 7.78]	2.33	1.38	0.167
	HR 5	10.36 [6.14, 14.57]	2.15	4.81	< 0.001
	HR 6	8.14 [4.24, 12.05]	1.99	4.09	< 0.001
	HR 7	3.69 [0.24, 7.15]	1.76	2.10	0.036
	HR 8	2.43 [-1.20, 6.06]	1.85	1.31	0.190

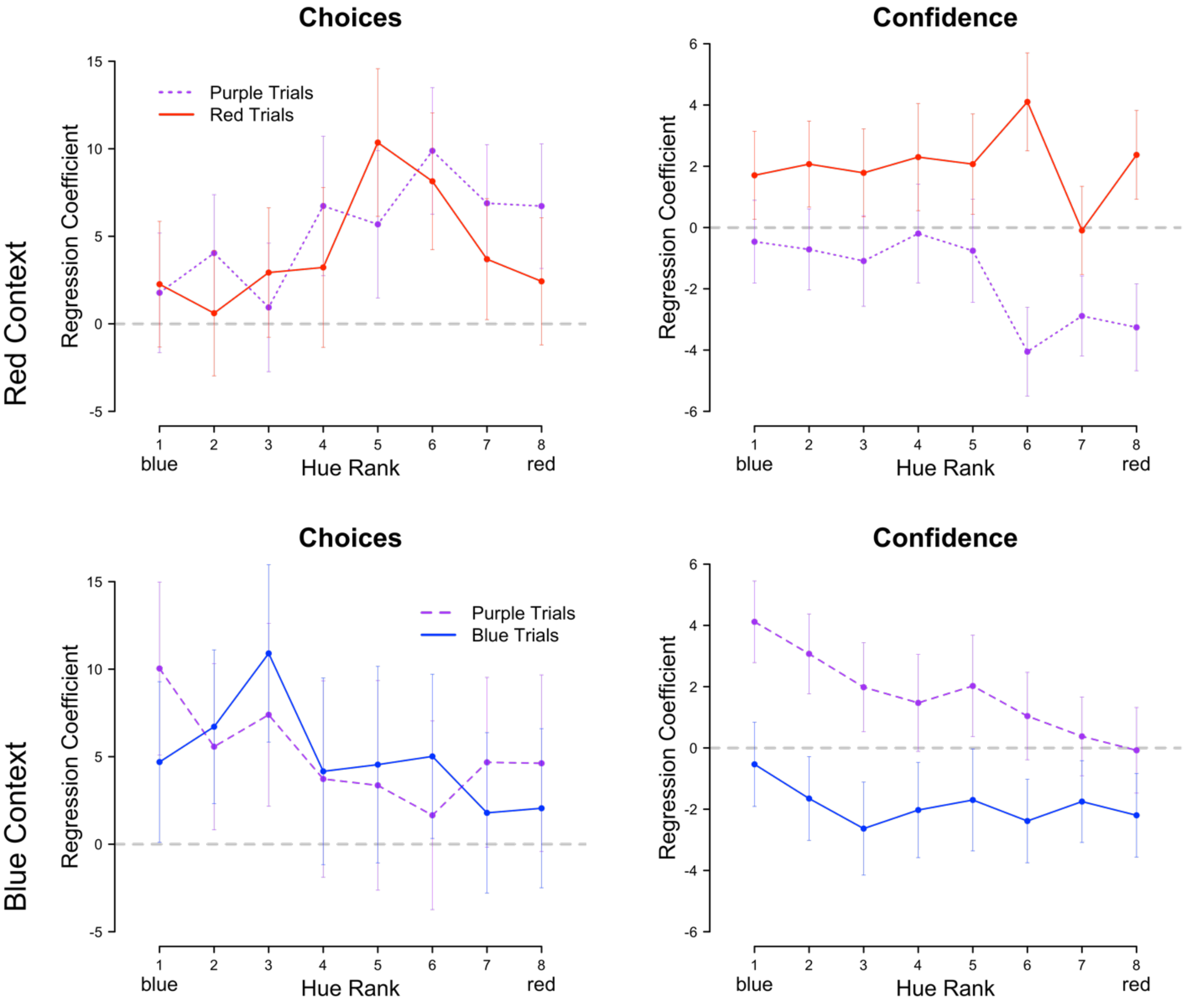

Figure S8. Reliance effects for choices and confidence from an additional, preregistered experiment. *The left column depicts choice reliance effects (how much choice could be predicted by each of the sorted, colored elements) and the right column confidence reliance effects (how much confidence could be predicted by each of the sorted, colored elements). The data are shown as a function of their stimulus category (line colors) and the color contexts in which they were shown (top row: Red Context; bottom row: Blue Context). Uncertainty is reflected in the error bars (2.5% and 97.5% Wald confidence intervals).*

Hypothesis 3: In a similar approach, we predicted confidence using a linear mixed model (see Table S3 and Figure S8, right panels). We expected to find an effect of congruency (Hypothesis 3a), which was indeed the case for the purple conditions (purple in the Blue Context: $\beta = 7.28$, SE = 2.29, $z = 3.18$, $p = 0.001$, 95% CI [2.80, 11.76]; purple in the Red Context: $\beta = 8.49$, SE = 2.33, $z = 3.65$, $p < 0.001$, 95% CI [3.93, 13.05]), but neither for the red, $\beta = -0.59$, SE = 2.54, $z = -0.23$, $p = 0.817$, 95% CI [-5.56, 4.39], nor blue trials, $\beta = 1.19$, SE = 2.41, $z = 0.49$, $p = 0.622$, 95% CI [-3.54, 5.91]. We again calculated the equivalent linear contrast, which showed no significant pattern (red: $\beta = -0.55$, SE = 12.25, $z = -0.05$, $p = 0.96$, 95% CI [-24.57, 23.46]; blue: $\beta = 11.08$, SE = 11.63, $z = 0.95$, $p = 0.341$, 95% CI [-11.72, 33.88]). It should be noted that we did not preregister this analysis for the red and blue trials. The results for the purple trials are reported in the main text. For the inlying-vs-outlying contrast (Hypothesis 3b), only the red and blue conditions showed the expected pattern (red: $\beta = 4.21$, SE = 1.29, $z = 3.25$, $p = 0.0012$, 95% CI [1.67, 6.74]; blue: $\beta = 2.61$, SE = 1.23, $z = 2.12$, $p = 0.034$, 95% CI [0.20, 5.02]), but not the other two (purple in Red Context: $\beta = -1.22$, SE = 1.24, $z = -0.99$, $p = 0.322$, 95% CI [-3.65, 1.20]; purple in Blue Context: $\beta = -0.97$, SE = 1.22, $z = -0.80$, $p = 0.425$, 95% CI [-3.36, 1.42]). We again deviated slightly from the preregistration by fitting four separate models due to multicollinearity concerns (cf. above, Hypothesis 2).

Table S3: Results from four linear mixed models predicting trial-by-trial confidence for the empirical data. HR = hue rank; CI = confidence interval; SE = standard error; df = degrees of freedom.

Model	Predictor	β [95% CI]	SE	df	*t*	*p*
Blue trials	Intercept	10.61 [8.67, 12.55]	0.99	4758.7	10.72	< 0.001
	HR 1	-0.53 [-1.91, 0.84]	0.70	4728.9	-0.76	0.446
	HR 2	-1.65 [-3.02, -0.29]	0.70	4728.8	-2.37	0.018
	HR 3	-2.63 [-4.15, -1.11]	0.77	4728.6	-3.40	0.001
	HR 4	-2.03 [-3.58, -0.47]	0.79	4728.8	-2.56	0.011
	HR 5	-1.70 [-3.36, -0.04]	0.85	4728.4	-2.00	0.045
	HR 6	-2.39 [-3.75, -1.02]	0.70	4728.2	-3.43	0.001
	HR 7	-1.75 [-3.09, -0.42]	0.68	4729.3	-2.57	0.010
	HR 8	-2.20 [-3.56, -0.84]	0.70	4728.7	-3.16	0.002
Purple trials in blue context	Intercept	-0.65 [-3.16, 1.85]	1.28	4761.0	-0.51	0.609
	HR 1	4.12 [2.79, 5.45]	0.68	4731.9	6.07	< 0.001
	HR 2	3.07 [1.77, 4.38]	0.66	4731.5	4.62	< 0.001
	HR 3	1.98 [0.53, 3.44]	0.74	4731.2	2.68	0.008
	HR 4	1.47 [-0.11, 3.05]	0.81	4731.9	1.82	0.068
	HR 5	2.02 [0.37, 3.68]	0.84	4731.6	2.40	0.017
	HR 6	1.04 [-0.39, 2.47]	0.73	4731.7	1.43	0.153
	HR 7	0.38 [-0.91, 1.66]	0.66	4731.6	0.58	0.565
	HR 8	-0.08 [-1.47, 1.32]	0.71	4731.9	-0.11	0.913
Purple trials in red context	Intercept	11.44 [8.89, 13.99]	1.30	4744.2	8.80	< 0.001
	HR 1	-0.46 [-1.81, 0.89]	0.69	4716.1	-0.67	0.503
	HR 2	-0.71 [-2.04, 0.61]	0.67	4715.5	-1.06	0.290
	HR 3	-1.09 [-2.57, 0.38]	0.75	4715.1	-1.45	0.147
	HR 4	-0.20 [-1.80, 1.41]	0.82	4716.0	-0.24	0.812
	HR 5	-0.76 [-2.44, 0.93]	0.86	4715.7	-0.88	0.378

	HR 6	-4.05 [-5.50, 2.61]	0.74	4715.7	-5.49	< 0.001
	HR 7	-2.89 [-4.20, 1.58]	0.67	4715.6	-4.33	< 0.001
	HR 8	-3.26 [-4.67, -1.84]	0.72	4716.0	-4.51	< 0.001
Red trials	Intercept	-4.19 [-7.17, -1.20]	1.52	4745.0	-2.75	< 0.001
	HR 1	1.71 [0.27, 3.14]	0.73	4723.9	2.33	0.020
	HR 2	2.07 [0.67, 3.48]	0.72	4724.5	2.89	0.004
	HR 3	1.79 [0.35, 3.22]	0.73	4723.2	2.44	0.015
	HR 4	2.30 [0.55, 4.05]	0.89	4723.5	2.58	0.010
	HR 5	2.07 [0.43, 3.71]	0.84	4723.9	2.48	0.013
	HR 6	4.10 [2.50, 5.70]	0.81	4723.7	5.03	< 0.001
	HR 7	-0.10 [-1.54, 1.34]	0.73	4724.0	-0.13	0.897
	HR 8	2.37 [0.92, 3.82]	0.74	4724.1	3.21	0.0013

Hypothesis 4a is reported in the main text. We did not carry out the tests for Hypothesis 4b, as in line with our preregistration, these were only planned if no effect had been found in the analysis of all available data (Hypothesis 4a). Furthermore, although two additional analyses were included in the preregistration document, we opted not to report them here, as they were more exploratory in nature and, given the already substantial length of the paper, would have added disproportionate detail.

Table S4: Results from two logistic mixed models predicting trial-by-trial choices for the empirical data. SE = standard error; HR = hue rank; CI = confidence interval. Due to the small and unscaled range of the predictors, odds ratios are not directly interpretable and therefore not included.

Model	Predictor	β [95% CI]	SE	*z*	*p*
Blue trials	Intercept	-16.47 [-17.43, -15.51]	0.49	-33.57	< 0.001
	HR 1	-1.73 [-2.36, -1.11]	0.32	-5.46	< 0.001
	HR 2	2.57 [1.62, 3.52]	0.48	5.32	< 0.001
	HR 3	4.99 [3.90, 6.08]	0.56	8.97	< 0.001
	HR 4	7.34 [6.20, 8.48]	0.58	12.59	< 0.001
	HR 5	6.44 [5.32, 7.56]	0.57	11.27	< 0.001
	HR 6	5.69 [4.64, 6.73]	0.54	10.63	< 0.001
	HR 7	3.73 [2.83, 4.63]	0.46	8.13	< 0.001
	HR 8	2.20 [1.58, 2.83]	0.32	6.96	< 0.001
Red trials	Intercept	-15.00 [-16.14, -13.86]	0.58	-25.78	< 0.001
	HR 1	3.38 [2.73, 4.02]	0.33	10.33	< 0.001
	HR 2	4.29 [3.37, 5.22]	0.47	9.14	< 0.001
	HR 3	7.05 [5.99, 8.12]	0.54	12.95	< 0.001
	HR 4	6.66 [5.54, 7.78]	0.57	11.63	< 0.001
	HR 5	5.39 [4.25, 6.53]	0.58	9.26	< 0.001
	HR 6	5.44 [4.32, 6.56]	0.57	9.52	< 0.001
	HR 7	1.45 [0.47, 2.42]	0.50	2.90	0.004
	HR 8	-1.31 [-1.95, -0.67]	0.33	-4.00	< 0.001

Table S5: Results from two linear mixed models predicting trial-by-trial confidence for the empirical data. HR = hue rank; CI = confidence interval; SE = standard error; df = degrees of freedom.

Model	Predictor	β [95% CI]	SE	df	*t*	*p*
Blue trials	Intercept	10.01 [9.61, 10.40]	0.20	123	49.44	< 0.001
	HR 1	0.63 [0.36, 0.89]	0.14	40908	4.65	< 0.001
	HR 2	-0.44 [-0.85, -0.02]	0.21	40895	-2.07	0.038
	HR 3	-1.47 [-1.94, -0.99]	0.24	40896	-6.05	< 0.001
	HR 4	-2.03 [-2.51, -1.54]	0.25	40902	-8.18	< 0.001
	HR 5	-2.11 [-2.57, -1.64]	0.24	40902	-8.92	< 0.001
	HR 6	-1.92 [-2.34, -1.50]	0.21	40906	-8.97	< 0.001
	HR 7	-1.51 [-1.86, -1.15]	0.18	40903	-8.35	< 0.001
	HR 8	-1.96 [-2.20, -1.72]	0.12	40923	-15.78	< 0.001
Red trials	Intercept	-1.08 [-1.50, -0.65]	0.22	469	-4.97	< 0.001
	HR 1	2.21 [1.97, 2.45]	0.12	40958	18.17	< 0.001
	HR 2	1.83 [1.49, 2.18]	0.18	40938	10.44	< 0.001
	HR 3	2.76 [2.35, 3.16]	0.21	40944	13.36	< 0.001
	HR 4	2.05 [1.60, 2.50]	0.23	40937	8.95	< 0.001
	HR 5	1.36 [0.88, 1.83]	0.24	40932	5.62	< 0.001
	HR 6	1.53 [1.06, 2.00]	0.24	40926	6.43	< 0.001
	HR 7	0.41 [0.005, 0.82]	0.21	40917	1.98	0.047
	HR 8	-0.45 [-0.71, -0.19]	0.13	40891	-3.41	< 0.001

Table S6: Results from planned linear contrasts testing for negative slopes in regression weights from two linear mixed models predicting confidence, conducted separately for blue and red trials in each data set. SE = standard error; CI = confidence interval; SE = standard error.

Model	Dataset	β [95% CI]	SE	*z*	*p*
Blue trials	1	43.95 [39.92, 47.98]	1.69	25.95	< 0.001
	2	11.22 [7.11, 15.33]	1.74	6.46	< 0.001
	3	42.90 [37.61, 48.19]	2.23	19.27	< 0.001
	4	46.76 [41.20, 52.32]	2.34	19.98	< 0.001
	5	18.85 [13.20, 24.51]	2.38	7.94	< 0.001
	6	18.38 [3.97, 32.80]	6.06	3.04	0.007
	7	15.91 [4.37, 27.45]	4.85	3.28	0.003
	8	34.31 [30.03, 38.60]	1.80	19.04	< 0.001
	9	5.11 [2.70, 7.52]	1.01	5.04	< 0.001
Red trials	1	45.10 [41.30, 48.90]	1.60	28.25	< 0.001
	2	20.63 [16.67, 24.59]	1.67	12.34	< 0.001
	3	45.17 [40.10, 50.23]	2.13	21.23	< 0.001
	4	49.64 [43.84, 55.44]	2.44	20.37	< 0.001
	5	23.11 [17.80, 28.41]	2.23	10.38	< 0.001
	6	64.67 [50.59, 78.76]	5.92	10.93	< 0.001
	7	24.52 [11.52, 37.52]	5.47	4.48	< 0.001
	8	42.00 [37.57, 46.43]	1.86	22.53	< 0.001
	9	9.08 [6.67, 11.50]	1.01	8.95	< 0.001

Table S7: Results from planned linear contrasts testing for congruency in regression weights from two linear mixed models predicting confidence, conducted separately for blue and red trials in each data set. CI = confidence interval; SE = standard error.

Model	Dataset	β [95% CI]	SE	*z*	*p*
Blue trials	1	10.04 [7.73, 12.34]	0.97	10.38	< 0.001
	2	1.25 [-0.15, 2.65]	0.59	2.11	0.095
	3	8.89 [5.58, 12.20]	1.39	6.38	< 0.001
	4	10.38 [7.03, 13.73]	1.41	7.36	< 0.001
	5	4.73 [1.17, 8.29]	1.50	3.16	0.005
	6	3.96 [-4.43, 12.36]	3.53	1.123	0.583
	7	6.54 [-0.44, 13.52]	2.94	2.23	0.073
	8	5.44 [2.97, 7.91]	1.04	5.23	< 0.001
	9	0.29 [-1.15, 1.72]	0.60	0.47	0.95
Red trials	1	8.78 [6.53, 11.03]	0.95	9.27	< 0.001
	2	3.60 [2.30, 4.90]	0.55	6.57	< 0.001
	3	9.16 [6.09, 12.22]	1.29	7.12	< 0.001
	4	13.85 [10.54, 17.15]	1.39	9.96	< 0.001
	5	5.70 [2.10, 9.30]	1.51	3.77	< 0.001
	6	11.67 [3.41, 19.93]	3.47	3.37	0.002
	7	7.12 [-0.74, 14.98]	3.31	2.15	0.088
	8	11.33 [8.79, 13.87]	1.07	10.61	< 0.001
	9	2.60 [1.20, 4.00]	0.59	4.42	< 0.001

Table S8: Results from planned linear contrasts comparing AIC values across seven computational models, based on a linear mixed-effects model with participants nested within experiments. CI = confidence interval; SE = standard error; df = degrees of freedom.

Contrast	β [95% CI]	SE	df	*t*	*p*
Model 1 - Model 2	1.42 [0.65, 2.19]	0.39	1050	3.63	0.0003
Model 1 - Model 3	-1.65 [-2.42, -0.88]	0.39	1050	-4.22	<.0001
Model 1 - Model 4	2.06 [1.30, 2.83]	0.39	1050	5.28	<.0001
Model 1 - Model 5	-1.08 [-1.85, -0.31]	0.39	1050	-2.76	0.006
Model 1 - Model 6	-0.75 [-1.52, 0.02]	0.39	1050	-1.92	0.055
Model 1 - Model 7	-7.13 [-7.89, -6.36]	0.39	1050	-18.23	<.0001
Model 2 - Model 3	-3.07 [-3.84, -2.30]	0.39	1050	-7.85	<.0001
Model 2 - Model 4	0.65 [-0.12, 1.41]	0.39	1050	1.65	0.0995
Model 2 - Model 5	-2.50 [-3.26, -1.73]	0.39	1050	-6.39	<.0001
Model 2 - Model 6	-2.17 [-2.94, -1.40]	0.39	1050	-5.55	<.0001
Model 2 - Model 7	-8.54 [-9.31, -7.78]	0.39	1050	-21.86	<.0001
Model 3 - Model 4	3.71 [2.95, 4.48]	0.39	1050	9.50	<.0001
Model 3 - Model 5	0.57 [-0.20, 1.34]	0.39	1050	1.46	0.145
Model 3 - Model 6	0.90 [0.13, 1.67]	0.39	1050	2.30	0.022
Model 3 - Model 7	-5.48 [-6.24, -4.71]	0.39	1050	-14.01	<.0001
Model 4 - Model 5	-3.14 [-3.91, -2.37]	0.39	1050	-8.04	<.0001
Model 4 - Model 6	-2.81 [-3.58, -2.05]	0.39	1050	-7.20	<.0001
Model 4 - Model 7	-9.19 [-9.96, -8.42]	0.39	1050	-23.50	<.0001
Model 5 - Model 6	0.33 [-0.44, 1.10]	0.39	1050	0.84	0.402
Model 5 - Model 7	-6.05 [-6.82, -5.28]	0.39	1050	-15.47	<.0001
Model 6 - Model 7	-6.38 [-7.14, -5.61]	0.39	1050	-16.31	<.0001

Table S9: Results from two linear mixed models predicting trial-by-trial confidence for the simulated data from the winning model (Model 4). HR = hue rank; CI = confidence interval; SE = standard error; df = degrees of freedom.

Model	Predictor	β [95% CI]	SE	df	*t*	*p*
Blue trials	Intercept	4.42 [4.30, 4.54]	0.06	8.48	72.62	< 0.001
	HR 1	0.51 [0.40, 0.62]	0.05	349300	9.26	< 0.001
	HR 2	-0.98 [-1.15, -0.82]	0.08	349900	-11.77	< 0.001
	HR 3	-1.67 [-1.85, -1.49]	0.09	350000	-17.87	< 0.001
	HR 4	-2.39 [-2.57, -2.21]	0.09	350000	-25.51	< 0.001
	HR 5	-3.54 [-3.71, -3.36]	0.09	350000	-39.55	< 0.001
	HR 6	-3.15 [-3.31, -2.99]	0.08	350100	-39.08	< 0.001
	HR 7	-1.98 [-2.11, -1.84]	0.07	350000	-28.63	< 0.001
	HR 8	-1.33 [-1.42, -1.23]	0.05	350100	-27.13	< 0.001
Red trials	Intercept	4.43 [4.31, 4.55]	0.06	8.40	71.52	< 0.001
	HR 1	1.25 [1.15, 1.34]	0.05	350100	25.59	< 0.001
	HR 2	2.05 [1.91, 2.18]	0.07	350100	29.76	< 0.001
	HR 3	3.17 [3.01, 3.32]	0.08	350100	39.3	< 0.001
	HR 4	3.41 [3.24, 3.59]	0.09	350000	38.25	< 0.001
	HR 5	2.35 [2.16, 2.53]	0.09	350000	24.9	< 0.001
	HR 6	1.69 [1.51, 1.87]	0.09	350000	18.09	< 0.001
	HR 7	1.05 [0.89, 1.22]	0.08	349900	12.61	< 0.001
	HR 8	-0.59 [-0.70, -0.48]	0.05	349400	-10.82	< 0.001

Supplementary References

Bartoń, K. (2023). MuMIn: Multi-model inference. *R Package Version 1.47.5*.
